# Krisenreaktion zu Beginn der COVID-19-Pandemie in Sammelunterkünften für Geflüchtete: Analyse der akteursübergreifenden Zusammenarbeit aus Perspektive der Aufnahmebehörden in Deutschland

**DOI:** 10.1007/s00103-023-03745-w

**Published:** 2023-07-07

**Authors:** Andreas W. Gold, Kayvan Bozorgmehr, Louise Biddle, Clara Perplies, Eilin Rast, Rosa Jahn

**Affiliations:** 1 Sektion Health Equity Studies und Migration, Abteilung für Allgemeinmedizin und Versorgungsforschung, Universitätsklinikum Heidelberg, Heidelberg, Deutschland Im Neuenheimer Feld 130.3, 69120; 2 AG Bevölkerungsmedizin und Versorgungsforschung, Fakultät für Gesundheitswissenschaften, Universität Bielefeld, Bielefeld, Deutschland

**Keywords:** Infektionserkrankungen, Migration, Gemeinschaftseinrichtungen, Zusammenarbeit, Resilienz, Infectious diseases, Migration, Refugee accommodation, Cooperation, Resilience

## Abstract

**Hintergrund:**

Geflüchtete Menschen in Sammelunterkünften (SU) sind durch hohe Belegungsdichte und gemeinschaftlich genutzte Räume einem erhöhten SARS-CoV-2-Infektionsrisiko ausgesetzt. Unklar ist, mit welchen (organisationalen) Akteuren und in welcher Form die Aufnahmebehörden im Rahmen ihrer Krisenreaktion zur Eindämmung der COVID-19-Pandemie zusammenarbeiteten. Ziel des Beitrags ist es, die Zusammenarbeit zwischen Aufnahmebehörden und weiteren an der Unterbringung und Versorgung beteiligten Akteuren während der ersten Welle der COVID-19-Pandemie darzustellen und Empfehlungen für eine zukünftig verbesserte Krisenreaktion abzuleiten.

**Methoden:**

Datengrundlage bilden qualitative Interviews die im Zeitraum Mai–Juli 2020 mit Ansprechpersonen in Aufnahmebehörden, die für die Unterbringung von Geflüchteten zuständig sind (*N* = 46). Es erfolgen eine Visualisierung von Akteursnetzwerken und eine qualitative Analyse des Datenmaterials mittels Framework-Methode.

**Ergebnisse:**

Die Aufnahmebehörden arbeiteten mit einer Vielzahl weiterer (organisationaler) Akteure zusammen. Am häufigsten wurden Gesundheitsämter, Sozialarbeiter*innen und Sicherheitsdienste genannt. Die Krisenreaktion fiel sehr unterschiedlich aus, häufig in Abhängigkeit von Engagement, Wissen und Einstellungen einzelner Personen und beteiligten Organisationen. Bei Abwesenheit einer koordinierenden Stelle konnte es zu Verzögerungen durch eine „Wartehaltung“ der beteiligten Akteure kommen.

**Fazit:**

Die Krisenreaktion in SU für Geflüchtete würde von einer klaren Zuordnung der koordinierenden Funktion an einen geeigneten Akteur profitieren. Anstelle von Ad-hoc-Lösungen bedarf es nachhaltiger Verbesserungen im Sinne einer transformativen Resilienz, um strukturelle Vulnerabilitäten zu reduzieren.

**Zusatzmaterial online:**

Zusätzliche Informationen sind in der Online-Version dieses Artikels (10.1007/s00103-023-03745-w) enthalten.

## Abstract (en)

**Background:**

Refugees in collective accommodation facilities are at increased risk of COVID-19-infections due to high occupancy density and shared spaces. It is unclear which (organisational) actors the reception authorities were working with in their crisis response and how. The aim of this paper is to examine the working arrangements between reception authorities and other actors involved in accommodation and (health) care during the first wave of the COVID-19 pandemic and to derive recommendations for future crisis responses.

**Methods:**

The analysis was based on qualitative interviews with representatives responsible for the reception and accommodation of refugees (*N* = 46) conducted from May to July 2020. Cross-actor networks were visualised, and a qualitative analysis of the data material was carried out using the framework method.

**Results:**

The reception authorities worked with a multitude of other (organisational) actors. Health authorities, social workers and security personnel were mentioned most frequently. The crisis response was found to be highly heterogeneous due to its dependence on the commitment, knowledge and attitude of the individuals and organisations involved. In the absence of a coordinating actor, there may also be delays due to a “wait-and-see” attitude of the actors involved.

**Conclusion:**

Crisis response in collective accommodation facilities for refugees would benefit from a clear allocation of the coordinating role to an appropriate actor. Instead of improvised ad hoc solutions, sustainable improvements in terms of transformative resilience are needed to reduce structural vulnerabilities.

## Hintergrund

Die COVID-19-Pandemie hat Gesundheitssysteme weltweit vor große Herausforderungen gestellt und verstärkt die Frage aufgeworfen, wie die medizinische Versorgung und der Gesundheitsschutz von Bevölkerungen auch in Krisensituationen aufrechterhalten werden können [1, 2]. Dies zeigt sich besonders deutlich bei der Versorgung von Geflüchteten, die in Erstaufnahmeeinrichtungen der Länder (EA) und in Gemeinschaftsunterkünften der Kreise und Kommunen (GU) leben [3, 4]. Häufig sind Geflüchtete in diesen Einrichtungen durch eine hohe Belegungsdichte, geteilte Zimmer und gemeinschaftlich genutzte Sanitäranlagen einem erhöhten Risiko für COVID-19 und andere Infektionserkrankungen ausgesetzt [5, 6]. Aufgrund fehlender flächendeckender Standards für die Unterbringung und medizinische Versorgung bestehen zwischen den Unterkünften ausgeprägte Unterschiede hinsichtlich Lage, Größe, Infrastruktur und Mindestumfang angebotener Leistungen. Spezifische Empfehlungen des Robert Koch-Instituts (RKI) zum Umgang mit COVID-19 in Sammelunterkünften für Geflüchtete^1^ (SU) wurden nach der ersten Pandemiewelle im Juli 2020 erstmals veröffentlicht [7].

Bereits präpandemisch war die Versorgung in SU geprägt von einer Vielfalt an Akteuren mit unterschiedlichen Zuständigkeiten, Rollen und Aufgaben [8–10]. Eine Evaluation der in der ersten Pandemiewelle in 8 Bundesländern (BL) ergriffenen Maßnahmen zeigte große Unterschiede in den Reaktionen auf die Pandemie bei gleichzeitig hoher Belastung der Aufnahmebehörden und weiterer Akteure in den SU [11]. Die akteursübergreifende Zusammenarbeit ist in Krisenzeiten besonders wichtig, um die verschiedenen Beteiligten in eine gemeinsame Gesamtstrategie zu integrieren [11]. Auch die internationale Literatur zur Resilienz von Gesundheitssystemen verweist auf den hohen Stellenwert der Zusammenarbeit um mit Unsicherheiten umzugehen, relevantes Wissen im Krisenfall zu erhalten und eine adäquate gemeinsame Krisenreaktion zu finden [12, 13]. Bislang fehlen jedoch tiefergehende Analysen dieser Aspekte im Kontext der COVID-19-Pandemie. Vor allem in fragmentierten Settings mit multiplen Organisationen, Akteuren und Zuständigkeiten stellt sich die Frage, welche Akteure in einer Krise zusammenarbeiten und wie sich dies ausgestaltet [14].

In der vorliegenden Arbeit soll die Zusammenarbeit zwischen den Aufnahmebehörden und weiteren an der Unterbringung und Versorgung beteiligten (organisationalen) Akteuren während der ersten Welle der COVID-19-Pandemie genauer untersucht und Empfehlungen für eine zukünftig verbesserte Krisenreaktion abgeleitet werden. Dabei wird mittels einer Visualisierung von Akteursnetzwerken und einer qualitativen Datenanalyse folgenden Fragestellungen nachgegangen:

- Mit welchen Akteuren arbeiteten die Aufnahmebehörden bei Maßnahmen zur Bewältigung der ersten Welle der COVID-19-Pandemie in SU für Geflüchtete zusammen?
- Welche Formen der Zusammenarbeit (Tab. 1) werden in diesem Kontext beschrieben?

**Table 1:** 

		**Horizontale Integration** (Auf der gleichen Hierarchieebene bzw. zwischen Akteuren mit gleichem Status)	
		**–**	**+**
**Vertikale Integration** (Über unterschiedliche Hierarchieebenen hinweg)	**+**	**Koordination** *Integration wird durch eine gemeinsame Managementhierarchie erreicht*	**Kooperation** *Integration basiert meist auf einem hierarchischen Management, aber in Verbindung mit freiwilligen Vereinbarungen und „gegenseitigen Anpassungen“ der beteiligten Organisationen*
	**–**	**Vertragsbasierte Zusammenarbeit** *Beteiligte Organisationen können auf einem Markt miteinander konkurrieren und dieser Wettbewerb kann zu einer Art von Integration durch vertragliche Beziehungen führen*	**Kollaboration** *Integration wird vorwiegend durch freiwillige Vereinbarungen und gegenseitige Anpassungen der beteiligten Organisationen erreicht*

## Methoden

Datengrundlage der hier vorgestellten Analyse sind semistrukturierte qualitative Leitfadeninterviews, die im Zeitraum Mai–Juli 2020 im Rahmen der Studie COVMIG (COVID-19 und Fluchtmigration: Situationsanalyse von Maßnahmen und Bedarfen in Aufnahmeeinrichtungen und Gemeinschaftsunterkünften in Deutschland) mit den für die Unterbringung Geflüchteter zuständigen unteren Aufnahmebehörden auf Kreisebene (GU; *n* = 30) bzw. den oberen Aufnahmebehörden auf Landesebene (EA; *n* = 16) in 8 BL geführt wurden. Der Leitfaden (*Onlinematerial 1*) beinhaltete wesentliche Dimensionen der Prävention und Gesundheitsförderung, Infektionskontrolle und akteursübergreifenden Zusammenarbeit. Die Interviews dauerten 25–105 min und wurden jeweils von 2 Forschenden geführt. Weitere Details können der Infobox entnommen werden und wurden bereits veröffentlicht [11]. Um die gewonnenen Daten mit Blick auf die Fragestellungen dieser Arbeit zu analysieren, wurde ein mehrschrittiges methodisches Vorgehen gewählt.

### Netzwerkanalyse

Zur Beantwortung der ersten Forschungsfrage wurde, orientiert an Methoden der sozialen Netzwerkanalyse [15, 16], eine Akteursmatrix auf Grundlage der qualitativen Interviewdaten mittels *MS Excel 2019* erstellt. Aus den Transkripten wurden unabhängig durch 2 Forschende (AG, RJ) alle Akteure extrahiert, mit denen eine Zusammenarbeit in relevanten Bereichen der Pandemiebewältigung erwähnt wurde. Etwaige Abweichungen wurden im Nachgang konsentiert. Informiert durch eine vorherige Analyse des Datenmaterials [11] wurden 5 Bereiche berücksichtigt: 1) prospektive *Konzepterstellung* zur Festlegung von Abläufen und Zuständigkeiten für vorhergesehene Eventualitäten, z. B. eines Nachweises von SARS-CoV‑2 in der Einrichtung, 2) *Aufklärung und Information* über pandemiebedingte Maßnahmen, 3) *Testung und Ermittlung von Kontaktpersonen*, 4) *Verlegung *von Risikopersonen und Entzerrung der Belegung zum Zwecke des Infektionsschutzes und 5) Vorbereitung und Durchführung von *Quarantäne und Isolation*.

Die Visualisierung erfolgte mittels Softwarepaket „gephi 0.9.2“ [17]. Für die Darstellung wurde ein klassischer Layout-Algorithmus nach Fruchterman-Reingold gewählt, bei dem sich Knotenpunkte (Nodes), die durch Linien (Edges) verbunden sind, gegenseitig anziehen [18]. Verbindungslinien zeigen Netzwerkkontakte der befragten Aufnahmebehörden zu anderen Akteuren an. In 2 der 46 Interviews machten die Befragten keinerlei Angabe zur Zusammenarbeit mit anderen Akteuren, entsprechend werden diese nicht visuell dargestellt. Erstellt wurden eine Gesamtdarstellung aller Akteure sowie Darstellungen der Zusammenarbeit zu den o. g. ausgewählten Bereichen. Die Positionen der einzelnen Akteure innerhalb der Visualisierung sind aus Gründen der Übersichtlichkeit in allen Abbildungen gleich und entsprechen den Positionen im Gesamtnetzwerk. Da Stabsstrukturen nicht als ausführende Akteure beschrieben wurden, sind sie in den Abbildungen nicht enthalten. Für eine bessere Übersicht werden Akteure bei vergleichbarer Funktion zusammengefasst sowie Akteursgruppen gebildet.

### Qualitative Analyse

Zur Beantwortung der zweiten Forschungsfrage erfolgte, unterstützt durch MAXQDA 2020 [19], eine vertiefende Analyse des umfangreichen qualitativen Datenmaterials. Hierfür konnte auf ein bereits für eine initiale Analyse [11] entwickeltes Codesystem aufgebaut werden (*Onlinematerial 1*). Informiert durch die entwickelten Visualisierungen des Datenmaterials wurden Formen der Zusammenarbeit zu den o. g. Themenbereichen mittels Frameworkmethode strukturiert analysiert [20].

Zur Typisierung unterschiedlicher Formen der Zusammenarbeit kommt das Framework von Axelsson und Axelsson (2006) zum Einsatz (Tab. 1). Hiernach können 4 Formen der Zusammenarbeit für die Integration der unterschiedlichen organisationalen Akteure in eine gemeinsame Gesamtstrategie differenziert werden: Kollaboration, Kooperation, Koordination und vertragsbasierte Zusammenarbeit [21]. Die Beschreibung im Ergebnisteil erfolgt anhand dieser Begrifflichkeiten. Ist eine Zuordnung aus dem Datenmaterial nicht möglich, wird allgemein von „Zusammenarbeit“ gesprochen.

## Ergebnisse

### Gesamtnetzwerk

**Figure 1:**
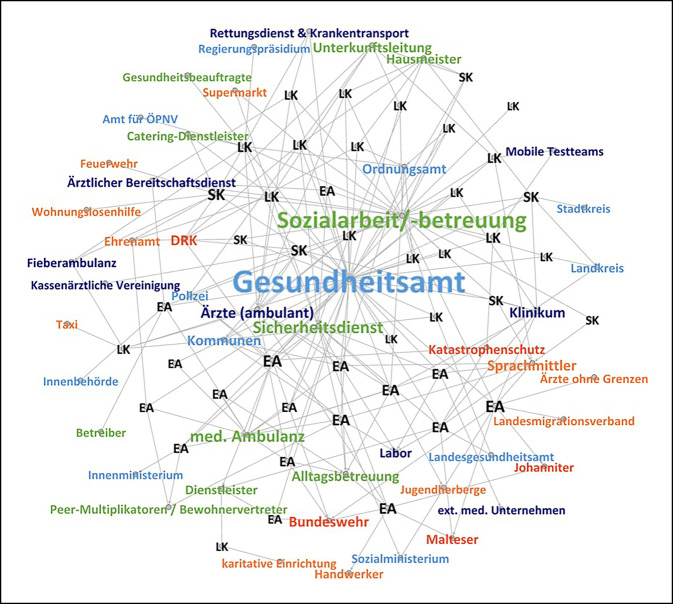

In 46 Interviews wurde die Zusammenarbeit mit insgesamt 48 unterschiedlichen (organisationalen) Akteuren berichtet (Abb. 1; tabellarisch: Onlinematerial 2). In 2 der Interviews wurden die Akteure, mit denen zusammengearbeitet wurde, nicht konkret benannt, diese Interviews sind daher von der grafischen Darstellung ausgeschlossen. Am häufigsten wurde eine Zusammenarbeit mit dem Gesundheitsamt (GA; *n* = 40), der Sozialarbeit/-betreuung (*n* = 28) und Sicherheitsdiensten (*n* = 14) beschrieben. Im medianen Mittel ($\tilde{x}$) berichteten Aufnahmebehörden eine Zusammenarbeit mit 5 unterschiedlichen Akteuren (min. = 0, max. = 11). Differenziert nach Setting (*Onlinematerial 3*) zeigt sich, dass in der Tendenz in EA ($\tilde{x}$ = 6; min. = 3, max. = 11) mit mehr Akteuren zusammengearbeitet wird als in GU ($\tilde{x}$ = 4; min. = 0, max. = 10).

Die dargestellten Akteure lassen sich in 5 Gruppen einteilen (Abb. 1). Eine Zusammenarbeit mit anderen *behördlichen Akteuren* erfolgte sowohl mit übergeordneten oder nachgelagerten behördlichen Strukturen (vertikal) als auch inner- und zwischenbehördlich (horizontal). Eine behördliche Zusammenarbeit fand dabei fast ausschließlich im unmittelbaren Tätigkeitsbereich und vorwiegend mit dem GA statt. Freigemeinnützige *Hilfsorganisationen* sind häufig auch außerhalb der Pandemie in Sammelunterkünften für Geflüchtete tätig, u. a. als Betreiber von EA oder auch als beauftragte Träger der Sozialarbeit. Im Pandemiekontext wurde zusätzlich eine Zusammenarbeit mit den Hilfsorganisationen in weiteren Funktionen (u. a. Rettungsdienst, Krankentransport, Katastrophenschutz) beschrieben.^2^ Eine Zusammenarbeit mit Strukturen des *Katastrophenschutzes* und der *Bundeswehr* kam v. a. bei größeren Ausbruchsgeschehen in Einrichtungen zum Tragen, um hierdurch den kurzfristigen Aufbau von Infrastruktur (u. a. zur Unterbringung und Testung) zu unterstützen. Deutliche Unterschiede zwischen den EA der Länder und den GU auf Stadt- und Landkreisebene (SK/LK) lassen sich v. a. in der Anzahl und Art der *einrichtungsbezogenen Akteure *feststellen. In EA sind aufgrund ihrer Funktion und der Unterkunftsgröße i. d. R. mehr Akteure unmittelbar vor Ort als in GU (Abb. 1). Unterkunftseigene medizinische Ambulanzen wurden z. B. ausschließlich für EA beschrieben und waren dort häufig unmittelbar in die Umsetzung von Eindämmungsmaßnahmen eingebunden. In den beteiligten GU wurde hingegen in der Regel auf eine Zusammenarbeit mit Strukturen der *medizinischen Routineversorgung* zurückgegriffen, beispielsweise für COVID-19-Testungen. Akteure, die sich keiner der vorgenannten Gruppen zuordnen lassen, werden unter *Sonstige* zusammengefasst.

Es zeigen sich erhebliche Unterschiede in Art, Umfang und Intensität der Zusammenarbeit mit anderen Akteuren. Eine Instanz zur *Koordination* von Maßnahmen zur Zielerreichung ist dabei in der Regel nicht explizit festgelegt. Bei Abwesenheit einer solchen Stelle zeigen sich unterschiedliche lokale Strategien, die von einer abwartenden Haltung in Hinblick auf zukünftige Vorgaben bis hin zu einem proaktiven Handeln im eigenen Arbeitsbereich oder sogar darüber hinaus reichen. Deutlich werden diese unterschiedlichen Strategien u. a. an Reaktionen auf das häufig als unzureichend erlebte Engagement des GA. Die meisten Interviewpartner*innen beschreiben zwar eine Zusammenarbeit mit dem GA, jedoch auch, dass diese im Ausmaß stark variiert [11]. Beispielsweise berichtete die Vertretung einer EA:

„Nachdem wir vom Gesundheitsamt keine Antwort bekommen haben, haben wir uns selber Gedanken gemacht und ein Konzept ausgearbeitet. Das haben wir dann wieder dem Gesundheitsamt geschickt und gefragt, ob das so recht wäre. Haben dann leider auch keine Antwort bekommen und dann alles so gemacht, wie wir das uns überlegt hatten“ (31EA).

Vor dem Hintergrund der hohen Akteursvielfalt sowie der häufig fehlenden oder gering ausgeprägten *koordinierenden* Stellen wurde die Integration der Aktivitäten zur Infektionskontrolle als herausfordernd beschrieben. Die Einbindung der Aufnahmebehörden in Krisen‑/Verwaltungsstäbe bzw. COVID-19-Taskforces (in 19 der 46 Interviews berichtet) wurde deshalb häufig positiv bewertet, da sie ein Forum für den strukturierten Informationsaustausch und zur gemeinsamen Planung von Maßnahmen boten:

„Ein Krisenstab ist eingerichtet worden und behandelt eigentlich alle Punkte. Nicht nur die Unterbringung, sondern ist im Prinzip Headquarter für diese Krise momentan“ (27EA).

### Konzepterstellung

**Figure 2:**
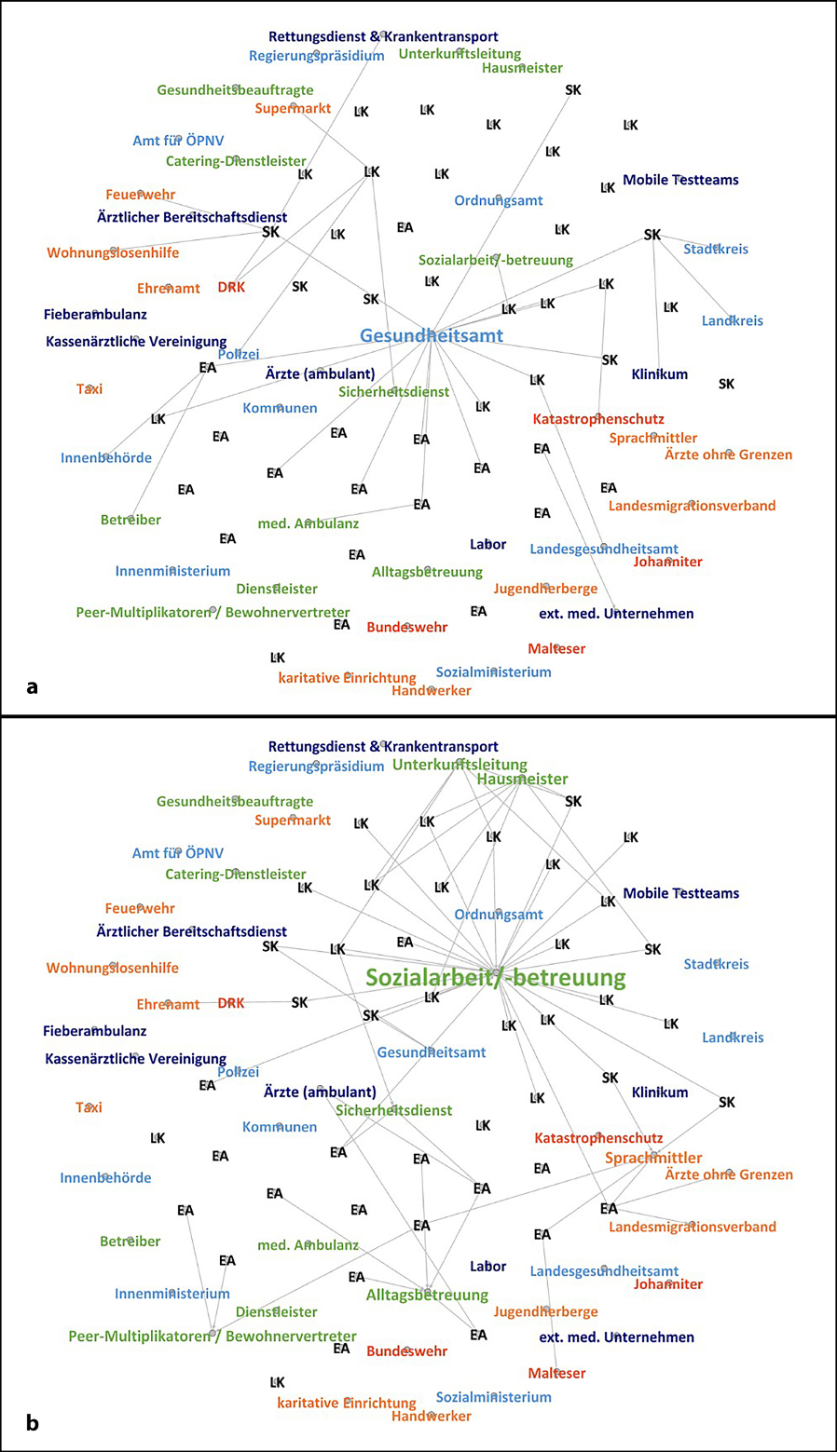

In 15 der 46 Interviews wurde eine Zusammenarbeit bei der Konzepterstellung berichtet (Abb. 2a). Dies wurde als besonders relevant für die Umsetzung von Maßnahmen bei bestätigten COVID-19-Fällen in der Einrichtung eingeschätzt, z. B. im Sinne von Ablaufplänen für Quarantänemaßnahmen und Testungen.

Hinsichtlich der Konzepterstellung wurde insbesondere eine *Kollaboration* mit oder *Koordination* durch das zuständige GA als wünschenswert beschrieben (*n* = 15). Gleichwohl zeigte sich eine eingeschränkte Responsivität der GA, was sowohl auf begrenzte Ressourcen und eine Überlastung des jeweils zuständigen GA als auch auf deren Einschätzung der Dringlichkeit einer konzeptionellen Zusammenarbeit zurückgeführt wurde. Als förderlich wurden feste Ansprechpartner*innen beim GA und ein persönliches Vertrauensverhältnis sowie eine frühzeitige proaktive Kontaktaufnahme beschrieben:

„Wir haben relativ früh begonnen uns Gedanken zu machen und so habe ich Ende Februar Kontakt mit unserem Gesundheitsamt aufgenommen. Als erstes haben wir ein Ablaufschema skizziert, wie wir bei Verdachtsfällen mit dem Gesundheitsamt vorgehen können“ (17LK).

Auch mit weiteren Akteuren wurden Abstimmungen zur Konzepterstellung berichtet, insbesondere wenn die Zusammenarbeit mit dem GA nicht zur gewünschten Klärung führte. Einzelne Einrichtungen arbeiteten mit anderen behördlichen Strukturen zusammen, teils wurde auch eine *Kooperation* mit Hilfsorganisationen und Katastrophenschutzstrukturen (*n* = 4) bei Ausbruchsgeschehen vorbesprochen (Abb. 2a). Ansprechpersonen aus der medizinischen Routineversorgung oder aus Ambulanzstrukturen in EA waren selten in konzeptionelle Abstimmungsprozesse eingebunden. Gleichwohl existierten in Einzelfällen Taskforces, die v. a. für den Aspekt Konzepterstellung auf *kollaborative* Formate der Zusammenarbeit setzten.

In 31 Interviews, in denen keine Zusammenarbeit bezüglich der Erstellung von Konzepten berichtet wurde, blieb dieser Arbeitsbereich entweder gänzlich unerwähnt (*n* = 9) oder es wurde trotz entsprechender Bemühungen keine Ansprechperson für eine Zusammenarbeit gefunden (*n* = 22). In diesen Fällen mussten die Aufnahmebehörden eigenständig Konzepte entwickeln, was aufgrund der fehlenden medizinischen Expertise als schwierig empfunden wurde:

„Ich bin kein Mediziner, ich bin Verwaltungsmitarbeiter. Es gibt da eben immer auch Unsicherheiten“ (24LK).

### Aufklärung und Information

In 24 der 46 Interviews wurde für eine persönliche Ansprache zur Aufklärung und Information der Bewohner*innen zu pandemiebedingten Maßnahmen mit Sozialarbeiter*innen und Alltagsbetreuer*innen zusammengearbeitet (Abb. 2b). Diese werden häufig durch Dienstleister bzw. freigemeinnützige Träger gestellt. Die Zusammenarbeit erfolgt damit formal *vertragsbasiert*, gleichwohl wurde in etlichen Interviews über eine eher *kooperative* oder *kollaborative* Zusammenarbeit mit freiwilligen Vereinbarungen und gegenseitigen Anpassungen der beteiligten Organisationen berichtet, die über vertragliche Vereinbarungen hinausgeht. In anderen Fällen ergab sich aus der externen Trägerschaft der Sozialarbeit aufgrund behördlicher Betretungsverbote oder durch Rückzug der Träger aus manchen Einrichtungen eine vorübergehende vollständige Aussetzung der Tätigkeiten vor Ort. In diesen Fällen erhielt das noch vor Ort verbliebene, allerdings fachfremde Personal (Unterkunftsleitung, Hausmeister, Sicherheitsdienst; *n* = 14) entweder explizit den Auftrag, Aufklärung und Information anzubieten, oder übernahm dies implizit:

„Erklärungen kommen von unserer Unterkunftsleitung und über die Sozialbetreuung. Und die Security, die auch einen Riesenauftrag hat, die Bewohner hinzuweisen oder zu erklären, warum man was machen soll oder nicht machen soll“ (21LK).

Eine Zusammenarbeit mit Vertreter*innen der medizinischen Routineversorgung oder des GA wurde hingegen selten benannt (*n* = 4), obwohl sie als besonders wirksam bewertet wurde:

„Wenn jemand kommt von einer Institution [Gesundheitsamt] – der ist auch kompetenter als wir Mitarbeiter vor Ort – hat das auch nochmal zusätzliches Gewicht. Das hat auch dazu beigetragen … dass die Maßnahmen besser akzeptiert wurden“ (2SK).

Eine *Kooperation* mit Vertreter*innen aus der Bewohnerschaft als Peer-Multiplikator*innen oder mit professioneller Sprachmittlung wurde nur von 7 Interviewpartner*innen (5 EA, 2 GU) beschrieben. Dies zukünftig zu intensivieren, wurde jedoch als eine wichtige Erkenntnis formuliert:

„Ich will in der Zukunft intensiv aufklären und das möglichst auch in allen Sprachen, damit ich auch den Letzten erreiche, … schon als vorbeugende Aufklärung“ (41EA).

### Testung und Ermittlung von Kontaktpersonen

**Figure 3:**
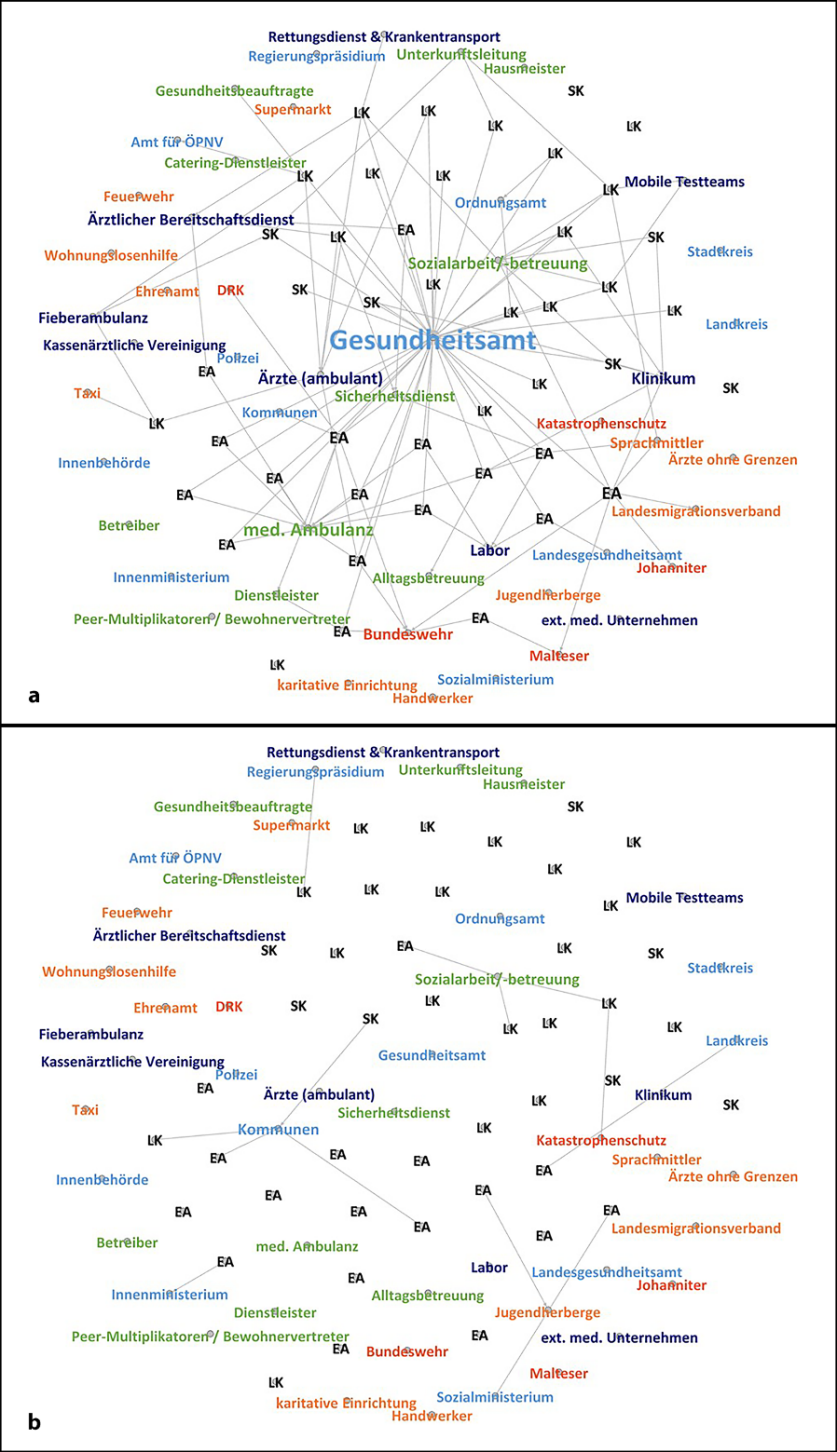

In 40 der 46 Interviews wurde eine Zusammenarbeit mit anderen Akteuren bei der Testung von Bewohner*innen und bei der Ermittlung von Kontaktpersonen beschrieben (Abb. 3a).^3^ Insgesamt zeigten sich eine hohe Heterogenität bei der Durchführung und entsprechende Unterschiede bei der Zusammenarbeit [11].

Für die Durchführung von Testungen war in der Regel eine Zusammenarbeit mit mehreren Akteuren Voraussetzung. Insbesondere in der Feststellung der Testberechtigung und Übermittlung von Testergebnissen übernahmen GA meist eine *koordinierende* Rolle, die auf entsprechenden vorgeschriebenen Verwaltungsverfahren beruhte. Die Informationsweitergabe war jedoch lokal unterschiedlich stark standardisiert und reichte von der Annahme, bei einem positiven Testergebnis wohl informiert zu werden, bis hin zu definierten Meldeketten. Eine Einbindung des GA in die Testdurchführung erfolgte nur in Einzelfällen:

„Ich *hoffe* doch, dass dann das Gesundheitsamt sich bei uns melden würde …“ (3SK).

„Wir haben natürlich dem Gesundheitsamt schon relativ früh alle Adressen gemeldet, sodass wenn im Gesundheitsamt ein COVID-19-Fall gemeldet wird, die das abgleichen und uns sofort informieren“ (26LK).

In EA führten in der Regel die medizinischen Ambulanzen die Testungen durch, hier konnte häufig auf eine bereits vorbestehende *Kooperation* aufgebaut werden. Bei größeren Ausbruchsgeschehen wurde eine Unterstützung bei der Probennahme durch Hilfsorganisationen (zivile Sanitätsdienste, teils auch im Rahmen der Katastrophenhilfe) oder den Sanitätsdienst der Bundeswehr berichtet. Einzelne EA schlossen zudem *Verträge *direkt mit Laboren, um Testauswertungen unabhängig von lokalen Gegebenheiten sicherstellen zu können. In GU konnte nicht auf medizinische Ambulanzen innerhalb der Einrichtungen zurückgegriffen werden. Häufig erfolgte die Testdurchführung über freiwillige *Kollaborationen* mit niedergelassenen Ärzt*innen oder über formalisierte *Kooperationen* mit mobilen Testteams unmittelbar vor Ort. In anderen Einrichtungen war dies hingegen nur in Arztpraxen oder Teststellen möglich und dadurch mit logistischem Aufwand für den Transport verbunden.

Die Kontaktpersonenermittlung bei bestätigten Fällen erfolgte häufig *kooperativ* in Abstimmung mit dem zuständigen GA, die praktische Durchführung übernahm jedoch meist das Personal der Einrichtungen. Als Gründe hierfür wurden sowohl der leichtere Zugang und bessere Überblick innerhalb der Einrichtungen, teils aber auch die ansonsten als zu lang empfundenen Bearbeitungszeiten genannt:

„Eigentlich muss man warten, bis uns das Gesundheitsamt sagt, wer in Quarantäne geht. Aber ich entscheide das als Bereichsleitung selber, bis das Gesundheitsamt mir einfach nur noch zustimmt“ (44EA).

Ein Einbezug von (qualifizierter) Sprachmittlung zur Aufklärung oder Kontaktpersonenermittlung wurde nur für einzelne Einrichtungen berichtet.

### Verlegung

In 11 der 46 Interviews wurde eine Zusammenarbeit mit weiteren Akteuren bei Verlegungen berichtet (Abb. 3b). Hierbei zeigten sich deutliche Unterschiede zwischen EA und GU. Für Verlegungen innerhalb der Einrichtungen berichteten manche EA über gewisse Reservekapazitäten aufgrund ihrer baulichen Struktur, die auch kurzfristig reaktiviert werden konnten. In GU bestanden solche Kapazitäten aufgrund landesrechtlicher Regularien meist nicht. Im Falle von Verlegungen innerhalb des eigenen Zuständigkeitsbereichs wurde am ehesten mit Sozialarbeiter*innen zusammengearbeitet.

Möglichkeiten zur Weiterverlegung in andere Einrichtungen variierten settingspezifisch. Die jeweils zuständigen Landesregierungen formulierten häufiger das politische Ziel, die Belegung in EA durch eine forcierte Weiterverlegung zu reduzieren. Verantwortliche der EA und der zuständigen ministeriellen Ebene arbeiteten hierzu *koordinierend* zusammen:

„Es sollten keine Transfers mehr stattfinden … Wir haben dann aber eine Ausnahme vom Ministerium bekommen, dass *wir* als einzige Einrichtung in [BL] unter bestimmten Voraussetzungen noch Transfers vornehmen dürfen, um unsere Belegung zu reduzieren“ (40EA).

Eine *Koordination* mit den aufnehmenden Kreisen und Kommunen wurde hingegen nur in Einzelfällen berichtet. Entsprechend bemängelten befragte GU-Vertreter*innen die ausbleibende Kommunikation, da die Verlegungen aus EA zu Kapazitätsengpässen in GU führten. Durch häufig bestehende Informationsdefizite (auch zum COVID-19-Status) und deutlich verkürzte Ankündigungsfristen wurde dies zusätzlich verstärkt:

„Was für uns tatsächlich derzeit das größte Problem ist, sind die Zuweisungen. … Wir kriegen, wenn wir Glück haben, drei Tage vorher gesagt, wer kommt und wie viele. Das läuft sehr unkoordiniert gerade“ (26LK).

Eine Weiterverlegung aus den GU in die nachgelagerten Kommunen war hingegen häufig eingeschränkt, vorbestehende *Kooperationsstrukturen* konnten dies erleichtern. In BL ohne bzw. mit ausgesetzter zentraler Verlegungssteuerung wurde teils auch über eine intensivierte *Kooperation* zwischen EA und aufnehmender Kommune berichtet. Als hilfreich wurde dabei ein unmittelbarer Kontakt zwischen entsendender und aufnehmender Stelle bewertet.

### Quarantäne und Isolation

**Figure 4:**
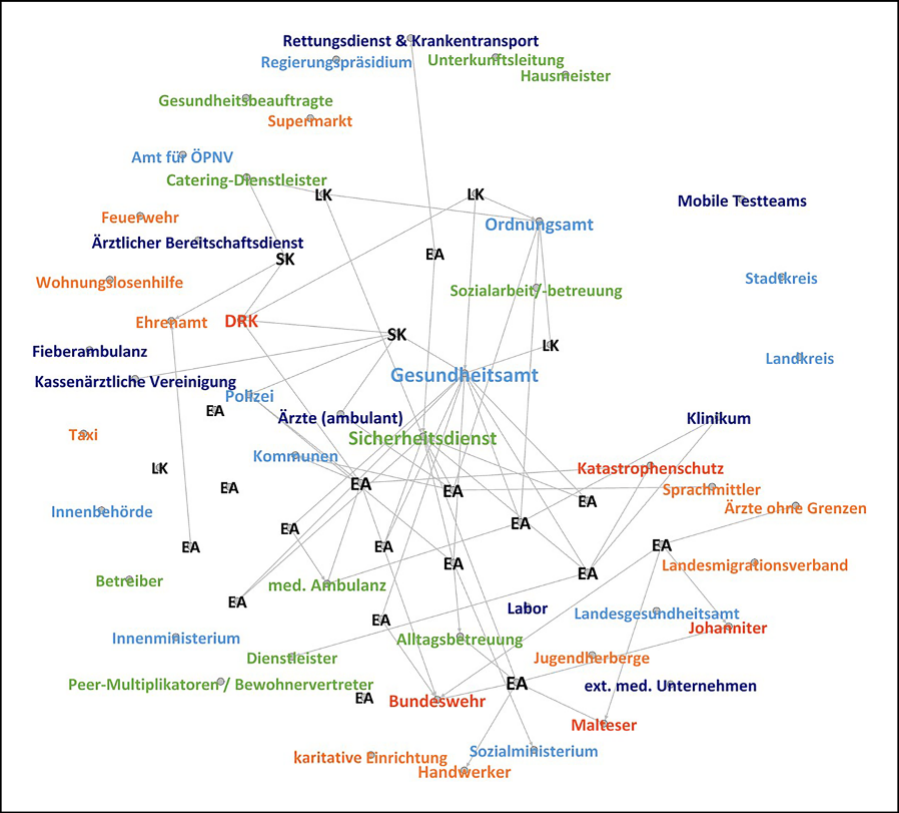

In 19 von 23 Interviews, in denen über bereits umgesetzte Quarantäne‑/Isolationsmaßnahmen berichtet wurde, wird eine diesbezügliche Zusammenarbeit mit anderen Akteuren angegeben (Abb. 4). Anknüpfungspunkte zu den Ordnungs- und Gesundheitsbehörden ergaben sich maßgeblich durch deren Zuständigkeit für die Anordnung der Maßnahmen. Bei der konkreten Umsetzung wurde insbesondere auf einrichtungsgebundene Akteure gesetzt, auch die gezielte Einbeziehung von Ehrenamtlichen wurde berichtet. Auffällig war eine verstärkte Beauftragung von Sicherheitsdiensten, während die medizinische und sozialarbeiterische Betreuung in den Hintergrund trat. In Einzelfällen wurde über die anlassbezogene Hinzuziehung der Polizei berichtet:

„Wir haben in vier Unterkünften von Anfang an Security gehabt und haben in der Coronasituation die Security auf ALLE Unterkünfte aufgestockt“ (21LK).

Im Rahmen größerer Ausbruchsgeschehen unterstützten mancherorts Hilfsorganisationen oder die Bundeswehr durch Aufbau von Infrastruktur und bei medizinischer Versorgung. Grundsätzlich zeigte sich in diesem Bereich die Notwendigkeit, häufig kurzfristig mit einer Vielzahl an unterschiedlichen und neu hinzukommenden Akteuren zusammenzuarbeiten. Die Aufnahmebehörden übernahmen dabei regelmäßig eine *koordinierende* Rolle, die jedoch mit einem erheblichen Aufwand verbunden war:

„Man muss gucken, dass da nichts schiefläuft. Drumherum stehen ganz viele Leute und die wollen alle alles wissen, und da ist die große Herausforderung, dass da nichts durchrutscht“ (15LK).

## Diskussion

Um auf die COVID-19-Pandemie adäquat reagieren und eine effektive Krisenreaktion sicherstellen zu können, sind die Aufnahmebehörden auf die Zusammenarbeit mit einer Vielzahl weiterer Akteure angewiesen. Am häufigsten werden das GA, Sozialarbeiter*innen und Sicherheitsdienste genannt. Unsere Analyse unterstreicht die große Akteursvielfalt in SU, mit der eine Differenzierung und Fragmentierung der Aufgaben in unterschiedlichen Bereichen der Pandemiebekämpfung einhergeht. Derzeit sind es vor allem kollaborative Formen der Zusammenarbeit, die eine zeitnahe gemeinsame Krisenreaktion sicherstellen. Diese *kollaborativen* Formate begünstigen jedoch gleichzeitig, dass das Ergebnis der Zusammenarbeit und die Maßnahmen in den einzelnen SU sehr unterschiedlich ausfallen und einer gewissen Zufälligkeit unterliegen, da die Krisenreaktion von Engagement, Wissen und Einstellungen einzelner Personen und Organisationen abhängt. In Abwesenheit eines *koordinierenden* Akteurs kann es zu einer Verzögerung der Krisenreaktion durch eine „Wartehaltung“ der Beteiligten kommen. Die häufig in der Krisensituation gefundenen Ad-hoc-Lösungen machen nachhaltige Verbesserungen im Sinne einer transformativen Resilienz praktisch unmöglich und schaffen strukturelle Vulnerabilitäten, die vermeidbar wären [22].

Die interorganisationale Zusammenarbeit in EA in Deutschland wurde bereits infolge des „Sommers der Migration“ 2015/2016 empirisch untersucht [23]. Es zeigten sich ebenfalls komplexe Akteursnetzwerke. Viele Herausforderungen wurden bereits in dieser und weiteren Analysen beschrieben, führten jedoch seitdem nicht zu einem nachhaltigen Strukturauf- und -umbau [23–25]. In Bezug auf COVID-19 werden diese Erkenntnisse auch durch ein internationales Scoping-Review zu Präventionsmaßnahmen in SU für Geflüchtete bestätigt [26]. Das Review zeigt Diskrepanzen zwischen internationalen Empfehlungen und der tatsächlichen Umsetzung von Maßnahmen in den Einrichtungen auf und führt diese auf einen Mangel an settingspezifischen Vorgaben von übergeordneter Ebene, unzureichende Möglichkeiten zur Vorbereitung auf Ausbruchsgeschehen in den Einrichtungen sowie auf fehlende Zusammenarbeit zurück [26].

Die Krisenreaktion in SU für Geflüchtete würde von einer klaren Zuordnung der *koordinierenden* Funktion zu einem geeigneten Akteur profitieren. So richten sich die erstmals am 10.07.2020 vom RKI veröffentlichten Empfehlungen an die lokalen Gesundheitsämter als zentrale Akteure [7]. Unsere Ergebnisse zeigen jedoch, dass die GA häufig *keine koordinierende* Rolle übernehmen und derzeit auch nicht ausreichend ausgestattet sind, um dies sicherstellen zu können [11, 27]. So kam laut den hier analysierten Interviews die Initiative zur prospektiven Konzepterstellung immer vonseiten der Aufnahmebehörden. Auch eine aktive Einbindung der GA in Aufklärungsmaßnahmen erfolgte nur in Einzelfällen. Um die in den Empfehlungen vorgesehenen Aufgaben übernehmen zu können, ist nebst der vielfach geforderten Stärkung der Strukturen des öffentlichen Gesundheitsdienstes [2, 27] vor allem eine klar kommunizierte Erweiterung bzw. Präzisierung ihres Aufgabenspektrums und Mandats im Kontext der Fluchtmigration unerlässlich.

Insbesondere wenn die Einbindung neuer Akteure notwendig wurde, wie zum Beispiel bei der Umsetzung von Isolations- und Quarantänemaßnahmen, zeigte sich die Relevanz effektiver und frühzeitiger Abstimmung von Zuständigkeiten und Abläufen. Bei kurzfristigem Einsatzbedarf für die Umsetzung von erstmals anfallenden Aufgaben werden *ad hoc* häufig fachfremde Akteure einbezogen. Dabei handelt es sich regelmäßig um Sicherheitspersonal, was zum Beispiel im Kontext von Quarantäne und Isolation zu einer „Versicherheitlichung“ beiträgt [23]. Durch regelmäßigen, strukturierten Austausch über Krisenstäbe oder enge persönliche Kontakte sowie durch frühzeitige konzeptionelle Abstimmung und Standardisierung konnte zwar in einigen Einrichtungen eine einheitliche Gesamtstrategie umgesetzt werden, aufgrund fehlender verbindlicher Vorgaben und entsprechender Mandate setzten diese Kollaborationen aber eine freiwillige Selbstverpflichtung und den „guten Willen“ der Beteiligten voraus.

Ebenfalls zentral ist es, die dargestellte Aufgaben‑, Akteurs- und Maßnahmenvielfalt vor dem Hintergrund der heterogenen und fragmentierten Unterbringungssituation in Deutschland zu betrachten. Daraus ergeben sich jeweils unterschiedliche strukturelle Bedingungen, die zu einer lokalen Differenzierung von Aufgabenspektren, Zuständigkeiten und Akteuren führen. Dies hat auch zur Folge, dass die für die Umsetzung von Infektionsschutzmaßnahmen gemäß § 36 Infektionsschutzgesetz (IfSG) verantwortliche Leitung einer Gemeinschaftseinrichtung nicht klar oder einheitlich definiert ist. Dies erschwert im Krisenfall die koordinative Integration der beteiligten Akteure und das Schaffen von einheitlichen Strukturen, die z. B. Synergien zwischen den Einrichtungen herstellen oder einen Austausch von Best Practices ermöglichen. Die heterogenen lokalen Bedingungen bedeuten zudem eine Herausforderung für das Verfassen von allgemeingültigen Empfehlungen für die Infektionskontrolle in SU, da diese einen Spielraum für lokale Anpassungen beinhalten und dennoch Mindestanforderungen formulieren müssen. Die *Koordination* einer übergreifenden Krisenreaktion in deutschen SU würde daher zusätzlich von einer Vereinheitlichung der Unterbringungsstrukturen und des Einrichtungsmanagements profitieren. Dies beinhaltet sowohl die Festlegung von Zuständigkeiten, behördliche Strukturen, digitale Lösungen, medizinische Versorgungsangebote und Sprachmittlung als auch Werkzeuge des Krisenmanagements wie feste Ansprechpersonen, dezidierte Quarantänemöglichkeiten und bei Bedarf aktivierbare Stabsstrukturen.

### Stärken und Schwächen

Die im Rahmen der Datenerhebung gewonnenen tiefen Einblicke in unterschiedliche Einrichtungen und die hohe Anzahl an Interviews aus verschiedenen BL stellen eine große Stärke der Studie dar. Die Visualisierung der Akteursnetzwerke ermöglicht einen neuartigen Zugang zum umfangreichen qualitativen Datenmaterial. Hiermit verbunden sind eine erleichterte Erkennung übergreifender Muster und Strukturen innerhalb des Datenmaterials und die Möglichkeit, darauf aufbauend weitere Forschungsfragen zur qualitativen Analyse zu generieren. Gleichwohl ist an dieser Stelle auch auf Schwächen hinzuweisen. So decken die Interviews lediglich die ersten Monate der Pandemie (05–07/2020) ab; die weitere dynamische Entwicklung des Pandemiegeschehens kann somit an dieser Stelle nicht mit abgebildet werden. Durch den geografischen Schwerpunkt bei der Durchführung der Interviews auf GU-Ebene auf ein Flächenland ist nicht auszuschließen, dass hierdurch bestimmte landesspezifische Strukturen überbetont sein könnten. Hinsichtlich der gewählten Analysemethode ist zu beachten, dass die Informationen für die Netzwerkanalyse aus qualitativem Datenmaterial auf Grundlage eines semistrukturierten Interviewleitfadens und nicht in Form einer dezidierten Abfrage gewonnen wurden. Gewisse Abweichungen zwischen den Interviews sind somit möglich. Grundsätzlich können die grafischen Darstellungen der Netzwerkanalyse die Quantität der Zusammenarbeit abbilden, jedoch nicht deren Qualität. Es bedarf daher der Kontextualisierung anhand des qualitativen Datenmaterials.

## Fazit

Die Auswertung zeigt, dass eine effektive Krisenreaktion auf die COVID-19-Pandemie in SU für Geflüchtete schwerpunktmäßig *kollaborativ* geprägt ist und damit grundlegend auf freiwilliger Selbstverpflichtung beruht. Dies ermöglicht zwar in einigen Einrichtungen die Entwicklung und Umsetzung einer einheitlichen Gesamtstrategie, ist aber stark von der Ressourcenausstattung, dem Engagement und der Haltung der beteiligten Akteure vor Ort abhängig. Dadurch ergibt sich eine Zufälligkeit in Ausmaß, Umsetzung und Qualität der vor Ort ergriffenen Maßnahmen des Infektionsschutzes, die der Resilienz des Gesundheits- und Aufnahmesystems in der Versorgung Geflüchteter im Weg steht und strukturelle Vulnerabilitäten schafft. Die Krisenreaktion in SU für Geflüchtete würde daher von einer klaren Zuordnung der koordinierenden Funktion an einen geeigneten Akteur sowie einheitlichen Standards des Einrichtungsmanagements, einschließlich der Festlegung der Einrichtungsleitung nach § 36 IfSG, profitieren.

### Infobox Datenerhebung und -aufbereitung im Rahmen der COVMIG-Studie [11], eigene Darstellung

*Datenerhebung:*

- Zeitraum der Datenerhebung: 01.05.–31.07.2020
- Rekrutierung zuständiger Ansprechpartner*innen in den Sozial‑, Migrations- oder Ordnungsämtern (GU) bzw. in den Landesbehörden (EA) zum Interview per schriftlicher Einladung:
  - Auf Ebene der EA wurden über ein überregionales Forschungsnetzwerk 18 Teilnehmer*innen aus 8 Bundesländern rekrutiert
  - Auf GU-Ebene wurden 27 Teilnehmer*innen in einem Flächenland mit einer hohen Flüchtlingszuweisung und großen regionalen Strukturunterschieden rekrutiert
  - Gezielte zusätzliche Rekrutierung von 3 Akteur*innen aus 3 weiteren Bundesländern zur Überprüfung auf mögliche Sättigungseffekte
- Durchführung semistrukturierter telefonischer Leitfadeninterviews durch je 2 Forschende
- Dauer der Interviews: 25–105 min

*Datenaufbereitung:*

- Digitale Aufzeichnung der Interviews (bei vorliegender Einwilligung), Transkription als Volltexte und Pseudonymisierung
- Aufbereitung der Daten mittels der Framework-Analysemethode [20] unterstützt durch MAXQDA Version 20 [19]:
  - Induktiv-deduktive Entwicklung eines Codesystems zu zentralen Themenbereichen
  - Erstellung einer Übersichtsmatrix mit relevanten Strukturmerkmalen, Informationen zu Mustern getroffener Maßnahmen und geäußerten Unterstützungsbedarfen für jeden Themenbereich

## Supplementary Information





## Footnotes

1 Hierunter werden EA und GU subsumiert.

2 In den visuellen Darstellungen erfolgt die Benennung anhand der Beschreibung der Befragten (namentliche Nennung oder ausgeübte Funktion).

3 In Interviews, in denen keine Zusammenarbeit berichtet wurde, wurden zum Zeitpunkt des Interviews auch keine bestätigten COVID-19-Fälle angegeben. Die Interviews mit diesen Einrichtungen wurden in KW 19/20 (2020) geführt.
