# Supplementary material for: Early crisis response to the COVID-19 pandemic in collective accommodation facilities for refugees: Analysis of cross-actor working arrangements from the perspective of the reception authorities in Germany
Source: Bundesgesundheitsblatt Gesundheitsforschung Gesundheitsschutz. 2023 Jul 7;66(8):890–900. [Article in German] doi: 10.1007/s00103-023-03745-w (PMC10371912; doi:10.1007/s00103-023-03745-w)
Supplement: Supplementary file 1 [file 103_2023_3745_MOESM1_ESM.pdf]

Onlinematerial 1

Semistrukturierter Leitfaden

1. Können Sie uns bitte kurz erläutern, wie Sie mit dem neuartigen Coronavirus in Ihren Aufnahmeeinrichtungen/ Gemeinschaftunterkünften umgehen?
2. Gibt es Landesspezifische / Landkreisspezifische Vorgaben zum Umgang und wenn ja welche?
3. Über welche Quellen beziehen Sie Ihre Informationen zum Umgang mit der Situation?
4. Wie erfahren Sie, dass es in Ihrer Unterkunft einen (potenziellen) COVID-19-Fall gibt? Was passiert dann?
5. Wie werden die Maßnahmen sozialer Distanzierung in den Unterkünften umgesetzt?
6. Wie erfolgen denn derzeit Angebote, die normalerweise in den Unterkünften gewährleistet werden, wie zum Beispiel die der Verfahrens- und Sozialberatung?
7. Wie informieren Sie die Bewohner über die Maßnahmen?
8. Mit welchen Akteuren auf Landes-/Landkreisebene arbeiten Sie im Zusammenhang mit der Versorgung Geflüchteter eng zusammen? Wie ist die Zusammenarbeit organisiert?
9. Wir haben jetzt verschiedene Punkte rund um den Umgang mit Covid-19 angesprochen. Gibt es aus Ihrer Sicht noch wichtige Punkte, die wir bisher noch nicht besprochen haben?

Codesystem zur Auswertung der Interviews (Oberkategorien)

1. Informieren von Bewohner*innen
2. Physische Distanzierung Bewohner*innen
3. Schutz- & Hygienemaßnahmen
4. Physische Distanzierung Mitarbeitende
5. Angebote
6. Intersektorale Zusammenarbeit
7. Gesundheitsversorgung
8. Testung
9. Eindämmungsmaßnahmen
10. Politischer Handlungsspielraum
11. Setting
12. Sonstiges allgemein

Onlinematerial 2

Anzahl der Interviews in denen eine Zusammenarbeit mit dem jeweiligen Akteur beschrieben wird, insgesamt und nach Bereichen der Zusammenarbeit (N=46 Interviews)

		Zusammenarbeit mit Akteur nach Bereichen				
**Akteur**	**Gesamt**	**Konzepterstellung**	**Aufklärung Bewohner*innen**	**Testung**	**Quarantäne**	**Verlegung**
Gesundheitsamt	40	15	2	38	12	0
Sozialarbeit/-betreuung	28	1	24	7	2	3
Sicherheitsdienst	14	1	3	4	10	0
med. Ambulanz	13	1	0	12	3	0
Ärzt*innen (ambulant)	11	0	2	6	3	0
Alltagsbetreuung	8	0	5	1	2	0
Kommunen	8	0	0	1	4	4
Klinikum	7	1	0	6	2	0
Sprachmittler*innen	7	0	4	3	2	0
Hausmeister*innen	6	0	6	0	0	0
Ordnungsamt	6	0	0	2	5	0
Unterkunftsleitung	6	0	5	3	0	0
Bundeswehr	5	0	0	5	4	0
DRK	5	1	0	1	4	0
Ehrenamt	5	0	2	0	3	0
Ärztlicher Bereitschaftsdienst	4	0	0	4	0	0
div. Dienstleister	4	0	0	2	2	0
Katastrophenschutz	4	2	0	0	2	1
Labor	4	0	0	4	0	0
Polizei	4	1	0	0	3	0
Catering-Dienstleister	3	0	0	0	1	0
Fieberambulanz	3	0	0	3	0	0

Handwerker*innen	3	0	0	0	3	0
Landkreis	3	1	0	0	1	1
Malteser	3	0	1	2	2	0
Peer-Multiplikator*innen / Bewohnervertreter*innen	3	0	3	0	0	0
Rettungsdienst & Krankentransport	3	1	0	1	1	0
Johanniter	2	0	0	1	2	0
Landesgesundheitsamt	2	1	0	1	0	0
Mobile Testteams	2	0	0	2	0	0
Sozialministerium	2	0	0	0	1	1
Supermarkt	2	1	0	0	1	0
Amt für ÖPNV	1	0	0	1	0	0
Ärzte ohne Grenzen	1	0	1	0	1	0
Betreiber	1	1	0	0	0	0
caritative Einrichtung	1	0	0	0	1	0
ext. med. Unternehmen	1	1	0	0	0	0
Feuerwehr	1	1	0	0	0	0
Gesundheitsbeauftragte	1	0	0	1	0	0
Innenbehörde	1	1	0	0	0	0
Innenministerium	1	0	0	0	0	1
Jugendherberge	1	0	0	0	0	1
Kassenärztliche Vereinigung	1	0	0	0	1	0
Landesmigrationsverband	1	0	1	1	0	0
Regierungspräsidium	1	0	0	0	0	1
Stadtkreis	1	1	0	0	0	0
Taxi	1	0	0	1	0	0
Wohnungslosenhilfe	1	1	0	0	0	0

Onlinematerial 3

Anzahl der genannten Akteure mit denen eine Zusammenarbeit beschrieben wird pro Interview, gesamt und nach Unterkunftsart (N=46 Interviews)

	**Median**	**Mittelwert**	**min**	**max**
Gesamt	5	5,2	0	11
Erstaufnahmeeinrichtungen (EA):	6	6,4	3	11
Gemeinschaftsunterkünfte Landkreis (LK):	4	4,2	0	10
Gemeinschaftsunterkünfte Stadtkreis (SK):	4,5	5,1	0	10
Gemeinschaftsunterkünfte gesamt (LK + SK):	4	4,4	0	10
